# Co-Ordination of Mucosal B Cell and CD8 T Cell Memory by Tissue-Resident CD4 Helper T Cells

**DOI:** 10.3390/cells10092355

**Published:** 2021-09-08

**Authors:** Young Min Son, Jie Sun

**Affiliations:** 1 Division of Pulmonary and Critical Medicine, Department of Medicine, Mayo Clinic, Rochester, MN 55905, USA; son.youngmin@mayo.edu; 2 Department of Immunology, Mayo Clinic, Rochester, MN 55905, USA; 3 Department of Physiology and Biomedical Engineering, Mayo Clinic, Rochester, MN 55905, USA; 4 Carter Immunology Center, University of Virginia, Charlottesville, VA 22908, USA; 5 Division of Infectious Disease and International Health, Department of Medicine, University of Virginia, Charlottesville, VA 22908, USA

**Keywords:** tissue resident memory T, tissue resident memory B, mucosal immunity, non-lymphoid tissues

## Abstract

Adaptive cellular immunity plays a major role in clearing microbial invasion of mucosal tissues in mammals. Following the clearance of primary pathogens, memory lymphocytes are established both systemically and locally at pathogen entry sites. Recently, resident memory CD8 T and B cells (T_RM_ and B_RM_ respectively), which are parked mainly in non-lymphoid mucosal tissues, were characterized and demonstrated to be essential for protection against secondary microbial invasion. Here we reviewed the current understanding of the cellular and molecular cues regulating CD8 T_RM_ and B_RM_ development, maintenance and function. We focused particularly on elucidating the role of a novel tissue-resident helper T (T_RH_) cell population in assisting T_RM_ and B_RM_ responses in the respiratory mucosa following viral infection. Finally, we argue that the promotion of T_RH_ responses by future mucosal vaccines would be key to the development of successful universal influenza or coronavirus vaccines, providing long-lasting immunity against a broad spectrum of viral strains.

## 1. Introduction

A cardinal feature of the adaptive immune system is the ability to develop immunological memory following primary antigenic encounter. Upon microbial invasion, naïve T cells, primed by antigen-presenting cells (APCs), rapidly undergo massive expansion and effector T cell differentiation to generate a large pool of antigen-specific effector T cells for the clearance of invading pathogens. Following pathogen clearance, effector T cells go through a contraction phase, in which majority of the effector T cells undergo apoptosis. The surviving effector cells or memory precursor cells convert into long-term memory T cells (including both CD4 and CD8 memory) after the contraction phase. Based on their trafficking properties, CD8 memory T cells can be further categorized into different subsets including central memory T (T_CM_), effector memory T (T_EM_), peripheral memory T (T_PM_) and tissue resident memory T (T_RM_) cells [1,2,3,4,5]. T_CM_ cells re-circulate through secondary lymphoid organs, T_EM_ cells have more broad capacity of mobility between blood and non-lymphoid tissues, while T_PM_ cells mainly patrol the blood vessels [4]. In contrast, T_RM_ cells are parked in non-lymphoid mucosal tissues and have an impaired capacity to enter re-circulation [6,7,8,9,10]. Following secondary infection with the same virus or viruses bearing conserved T cell epitopes, memory T cells are rapidly activated, undergo secondary effector T cell expansion and differentiation, and mediate prompt pathogen clearance before their systemic dissemination.

Similarly, naïve B cells can be activated and differentiated into extrafollicular plasmablasts (PBs) or germinal center (GC) B cells in secondary lymphoid organs. The primary extrafollicular PBs have been identified as short-lived antibody secreting cells and may provide a significant source of protective antibodies during microbial infections [11]. GCs are anatomically distinguished into two regions, the dark zone and the light zone [12]. Proliferation and somatic hypermutation of antigen-specific GC B cells occur in the dark zone, following which mutated B cell clones move into the light zone to terminate their differentiation. Within the light zone, B cells internalize antigens which are presented by follicular DCs (FDCs) and interact with follicular helper T (T_FH_) cells, a major CD4 T helper subset facilitating B cell-help [13,14]. GC B cells can further differentiate into long-lived plasma cells (LLPCs) or memory B cells (MBCs). LLPCs mediate long-term antibody secretion following primary infection, while MBCs can respond to secondary infection to either differentiate into PBs or re-enter GCs to undergo further affinity maturation [15,16,17]. Like memory T cells, MBCs can be divided into circulating and tissue-resident (B_RM_) populations, which reside in mucosal tissues [18].

Despite intensive studies on the cellular and molecular programming of memory lymphocyte generation over the past two decades, our understanding of the mechanisms of memory T and B cell maintenance and function, particularly in the mucosal tissues remains limited. Furthermore, little is known regarding the cellular and molecular pathways that may be targeted to simultaneously promote both B_RM_ and T_RM_ cell responses in the mucosal tissues. Here we will review the current understanding of mechanisms maintaining long-term immunological memory in mucosal tissues, with a focus on the roles of a subset of CD4 T helper cells, tissue-resident T helper cells (T_RH_), in coordinating respiratory mucosal CD8 and B cell memory responses following viral infection.

## 2. Tissue-Resident Memory T (T_RM_) and B (B_RM_) Cells

### 2.1. Generation of CD8 T_RM_ Cells

CD8 T_RM_ cell development is initiated when dendritic cells (DCs), either resident within lymph nodes or following migration from peripheral tissues, prime naïve T cells into effector CD8 T cells in draining lymph nodes. Antigen-experienced effector CD8 T cells subsequently infiltrate infected tissue to mediate pathogen clearance, after which a subset of CD8 T cells persists to become T_RM_ cells. Interestingly, migrating DCs can also precondition naïve T cells towards a resident memory T cell fate via the activation and presentation of transforming growth factor (TGF)-β [19]. Furthermore, distinct DC subsets appear to differentially program lymph node homing T_CM_, mucosal tissue-homing effector and resident memory T cells following influenza infection [20]. Therefore, T_RM_ cell development may start before the entry of effector T cells into peripheral organs. Such a notion is supported by recent genetic tracing (using retroviral barcoding) and single cell RNA-seq experiments demonstrating the existence of T_RM_ precursors in the circulating effector T cell pool [21].

After their activation in lymphoid organs, effector CD8 T cells enter the circulation and migrate into nonlymphoid tissues to combat invading pathogens. While CD8 T_RM_ fate determination can be trained in lymphoid organs, local environment and antigenic re-encounter in the tissue further promote T_RM_ development and/or maturation. The differentiation of T_RM_ cells can occur independently of local antigen recognition in the peripheral tissue [22,23,24]; however, local antigenic re-stimulation of effector CD8 T cells in nonlymphoid tissue greatly enhances T_RM_ formation [25,26,27]. Following the formation of T_RM_ cells, it is believed that T_RM_ maintenance is largely antigen or TCR signaling independent [28]. However, chronic low levels of TCR stimulation due to persistence of antigen following influenza virus infection or following immunization with an adenoviral vector facilitates the accumulation of a protective population of CD69^+^ CD8 T_RM_ cells [29,30].

CD69 is a key tissue retention signal of T_RM_ cells functioning via the interference of sphingosine-1-phosphate receptor (S1pr1) activity [31,32], thereby restricting cell egress out of the tissue [31,33]. Local antigen-restimulation enhances the expression of CD69, and concomitantly suppresses S1pr1 and Krüppel-like Factor 2 (KLF2) expression [31,34]. T_RM_ cells located within the epithelium further express CD103 (Integrin, alpha E), which binds to E-cadherin expressed on epithelial cells, supporting the accumulation and retention of T_RM_ cells in tissues [22,35,36]. In the absence of TGF-β signaling, the migrated tissue effector CD8 T cells fail to develop into CD103^+^ T_RM_ cells due to lack of CD103 expression [35,37,38]. Interleukin (IL)-15 has been reported to provide a survival signal to memory T cells [39]. Soluble IL-15/IL-15Rα complexes in local tissue do not directly induce CXCR3 production in effector cells, but promote the recruitment of CXCR3^+^ antigen-specific effector T cells to the mucosal area through the downregulation of KLF2 [40]. IL-7 is another important cytokine able to maintain memory T cell homeostasis through Stat5 signaling [41,42]. Interestingly, while IL-7 and IL-15 can both promote CD8 T_RM_ cell maintenance, the homeostatic persistence of skin CD4 T_RM_ cells seems mainly dependent on IL-7 produced from hair follicle of skin [43]. TGF-β has also been reported to facilitate CD8 T_RM_ cell development via down-regulation of T-box transcription factor Eomes and T-bet expression, but residual T-bet activity is critical to maintain responsiveness to IL-15 for CD8 T_RM_ cell survival [44]. In the lymphocytic choriomeningitis virus (LCMV) infection model, combination of cytokines including TGF-β, IL-33 and tumor necrosis factor (TNF) enhances the establishment of CD8 T_RM_ cells via the downregulation of S1pr1 [45]. Additionally, proinflammatory cytokines including type I interferons and IL-12 can facilitate the differentiation and accumulation of CD103^−^ T_RM_ cells in the intestine following bacterial infection [46]. Taken together, CD8 T_RM_ generation is constantly modulated by a variety of antigenic and environmental factors at various steps of the T cell life cycle following infection.

### 2.2. Transcriptional Regulation of CD8 T_RM_ Development and Persistence

T_RM_ cells exhibit distinct transcriptional profiles when compared to circulating memory T cells. Several transcription factors have been demonstrated to play important roles in T_RM_ cell generation and/or maintenance in peripheral tissues. The two related transcription factors, Blimp-1 and Hobit, cooperatively instruct a tissue-residency transcriptional program in T_RM_ cells [10]. Hobit and Blimp-1 directly bind to Klf2 and Tcf7 loci, and then downregulate the expression of Ccr7 and S1pr1. Therefore, they prevent the egress of T_RM_ cells from tissues to blood circulation [10,45]. Runx3 promotes the expression of CD103 and tissue residency-associated gene programs in T_RM_ cells, while simultaneously repressing signature genes associated with circulating memory [47]. Runx3 has been reported to enhance the accessibility of the Blimp1 binding region and may facilitate the accessibility of the Hobit binding motif as well [48]. Notch signaling through RBP-jκ is also important for the formation of lung T_RM_ cells following influenza virus infection [49]. Of note, TGF-β and Notch signaling can be integrated by direct protein–protein interactions of Smad3 and the intracellular domain of Notch (NICD), potentially providing a mechanism underlying the dual requirement of TGF-β and Notch in T_RM_ formation [50,51]. Compared to circulating memory T cells, T_RM_ cells highly express the transcription factor Bhlhe40 and its-associated molecules. Bhlhe40 deficiency caused diminished expression of T_RM_ tissue residency-associated genes and molecules involved with T_RM_ effector function, largely due to impaired mitochondria fitness and function in Bhlhe40-deficient T_RM_ cells [52]. Additionally, the expression of Ahr and NR4A1 is upregulated in T_RM_ cells compared to circulating memory CD8 T cells, and their function is required for the maintenance of CD8 T_RM_ cells [37,53,54].

### 2.3. Development and Maintenance of B_RM_ Cells

Long-term humoral immunity is generally maintained by LLPCs in the bone marrow (BM) [55], while resting MBCs provide rapid and augmented antibody (Ab) responses upon recognition of same/conserved antigens following secondary infections [56]. The development of both cell types is initiated within the GC structure following help from CD4 T cells [57,58]. Analogous to memory T cells, MBCs can be divided into circulating and tissue-resident (B_RM_) populations. B_RM_ cells, mainly residing in non-lymphoid peripheral tissues, express similar tissue-homing and retention molecules as T_RM_ cells. For instance, influenza hemagglutinin-specific lung-residing memory B cells [59] highly express CXCR3 and CD69, which are believed to mediate their tissue-homing and residency respectively [59]. In humans, a large number of CD19^+^CD27^+^CD45RB^+^CD69^+^ B_RM_ cells have been identified in the gut and tonsil but not in blood and BM [60].

Compared to circulating memory B cells, B_RM_ cells facilitate rapid recall responses in the tissue and may exhibit unique phenotypical and functional markers. For instance, mouse lung B_RM_ expresses lower CD73 than those MBCs in the blood or spleen [18]. Furthermore, respiratory B_RM_ cells reside in specific niches; in the upper respiratory tract, these are located in the nasal-associated lymphoid tissue (NALT) whereas they are found in the inducible bronchus associated lymphoid tissue (iBALT) in the lower respiratory tract [61,62,63]. Like T_RM_ cell development, B_RM_ cells are thought to be initiated following CD4 T cell help in secondary lymphoid organs, but the full establishment of B_RM_ cells in peripheral tissue requires local antigenic re-encounter [18]. Additionally, GC B cells of the lung, particularly those developed early post infection, may also supply B_RM_ precursors following influenza virus infection [64,65]. Thus, optimal B_RM_ development is subject to both distal regulation in the lymphoid organs and local regulation inside the peripheral tissue.

The functions of B_RM_ cells have not been fully elucidated yet. However, B_RM_ cells are thought to provide immediate and rapid responses against pathogen entry at mucosal tissues [66]. MBCs re-activated by pathogens have been reported to either differentiate into antibody secreting cells (ASCs) or undergo expansion and affinity maturation through re-entry of GC [67,68,69]. Lung influenza-specific B_RM_ cells are reported to directly differentiate into ASCs upon recognition of the same viral antigen during influenza reinfection, but do not re-enter the GC structure, thereby facilitating viral clearance in the respiratory mucosa [18]. Interestingly, influenza-specific lung B_RM_ cells developed from local GC responses possess high cross-reactivity to viral escape mutants [64,65], and thus they may exert broadly protective function against distinct viral strains. Furthermore, B_RM_ cells located in tertiary lymphoid structures are also considered potential APCs and may facilitate T cell responses in the respiratory mucosa [66,70]. In addition to influenza infection, antigen-specific B_RM_ cells are also developed following *pneumococcal* infection, although tertiary lymphoid organ (i.e., iBALT) formation was not observed. Importantly, depletion of PD-L2^+^ lung B_RM_ cells caused diminished bacterial clearance and reduction of *pneumococcus*-reactive antibodies in the lung upon pneumococcal reinfection, suggesting that lung B_RM_ cells are vital for pulmonary antibacterial immunity [71].

## 3. Characteristic Tissue-Resident CD4 T Cells

### 3.1. Heterogeneity of Tissue-Resident CD4 T Cells

The mechanisms underlying CD4 T_RM_ cell formation and maintenance are relatively less well-studied compared to those of CD8 T_RM_ cells. Similar to CD8 T_RM_ cells, activated CD4 T cells migrate into peripheral tissues and survive long term to form CD4 T_RM_ cells. CD4 T_RM_ cells share tissue-residency markers like CD69 and CXCR6 with CD8 T_RM_ cells [72,73]. Like mouse T_RM_ cells, human CD4 T_RM_ cells also express high level of CD69 while circulating CD4 memory T cells do not [73,74]. Unlike effector CD8 T cells, which mainly produce type 1 cytokines such as IFN-γ and TNF, effector CD4 T cells can be subdivided into distinct subtypes based on their cytokine production including IFN-γ producing T helper type 1 (T_H_1), IL-4/5/13-producing T helper type 2 (T_H_2), IL-17 producing T helper type 17 (T_H_17) and IL-21 producing follicular helper T (T_FH_) cells [75,76]. In the murine model, tissue-resident T_H_1 (T_H_1 T_RM_) cells have been reported following respiratory infection. Lung T_H_1 T_RM_ cells developed following influenza virus infection rapidly produce IFN-γ and contribute to host protection upon secondary infection [77,78]. T_H_1 T_RM_ cells have also been identified in the skin and gut following *Leishmania* and *Listeria* infections, respectively [79,80,81]. T_H_1 T_RM_ cells generated by *tuberculosis* are characterized by high expression of CXCR3 and low expression of KLRG1 [82,83]. For long-term maintenance in tissues, T_H_1 T_RM_ cells express high levels of CD11a and VLA-1 to promote their retention and survival in the tissue niche [84]. T_H_2 T_RM_ are usually generated during allergic responses or parasitic infections. In a house dust mite (HDM)-induced allergic asthma model, it was shown that lung T_RM_ cells and circulating T_H_2 memory cells cooperatively induce allergic inflammation in the lung [85]. During *Heligmosomoides polygyrus* infection, T_H_2 T_RM_ cells are formed and persist in the lamina propria and peritoneal cavity (PC). Interestingly, T_H_2 T_RM_ cells in both sites produce general T_H_2 cytokines like IL-4, IL-5 and IL-13 following TCR restimulation, but only T_H_2 T_RM_ in PC can respond with IL-33 and IL-7 upon TCR-independent restimulation [86]. T_H_17 T_RM_ can be generated following *Candida albicans* (*C. albicans*) infection in both mouse and human subjects. Both circulating T_H_17 memory cells and T_H_17 T_RM_ cells are important to clear the *C. albicans* upon rechallenge, but T_H_17 T_RM_ cells are more effective in rapidly clearing the pathogens [87]. In *Mycobacterium tuberculosis* (*M. tuberculosis*)-infected patients, lung CD4 T_RM_ cells produce IL-17 following antigenic stimulation, which along with IL-17 and IL-2 produced by T_H_17 T_RM_ cells suppress the growth of *M. tuberculosis* in 3D culture system [88].

Like effector T_RM_ subsets, Foxp-3 expressing regulatory T cells (T_REG_) cells in the tissue can express CD69 and possess tissue-residency features. Importantly, tissue-resident T_REG_ cells may provide an essential check point for the pathogenic activities of T_RM_ cells. Chronic exposure of *Aspergillus fumigatus* induces the formation of CD69^hi^CD103^lo^CD4 T_RM_ cells, which contribute to pulmonary fibrosis. At the same time, CD69^hi^CD103^hi^Foxp3^+^ T_REG_ cells constrain the effects of pathogenic CD103^lo^ CD4 T_RM_ cells and limit their fibrogenic potential [89]. Furthermore, lung tissue T_REG_ cells produce amphiregulin (Areg), an epidermal growth factor receptor ligand, to repair tissue damage following influenza virus infection [90].

Common gamma-chain cytokines such as IL-2, IL-15 and IL-7 are essential to develop or maintain memory CD4 T cells. The cytokines have recently been reported to be essential in the formation of CD4 T_RM_ cells. Autocrine IL-2 signaling in infiltrating tissue CD4 T cells is critical for the generation of T_H_1 T_RM_ cells in the lung [91]. In the absence of IL-2R signaling, T_H_1 T_RM_ cells induced by intranasal LCMV infection or allergic T_H_2 T_RM_ cells generated following HDM administration fail to be maintained over the long-term within the lung [92,93]. High levels of IL-7 receptor are expressed by lung T_RM_ cells compared to circulating memory T cells, and IL-7 treatment in vivo induces the infiltration of circulating CD4 T cells into the lung to form T_RM_ cells [94]. Similarly, IL-15 supports the development of lung CD4 T_RM_ post influenza virus infection [95].

### 3.2. Niches of Local CD4 T Cells

Tissue-resident CD4 T cells are typically located under epithelial layers and inside the ectopic lymphoid structures with stromal cells or APCs [96]. In the skin, particularly the dermis, lymphoid structures are generated around hair follicles with CD4 T cells and CD11b^+^DCs [97,98]. CCL5, IL-7 and IL-15 which are produced in the local environment, promote the maintenance of CD4 T_RM_ clusters in skin [43,97]. The female reproductive tract (FRT) is a mucosal tissue that consists of two different areas, including the upper FRT and the lower FRT. Mucosa-associated lymphoid tissues (MALT) composed of B cells and CD4 T_RM_ cells are typically developed in the lamina propria (LP) of the upper FRT. Furthermore, CD4 T cells can migrate to upper FRT following skin infection with *Chlamydia* to form a cluster with B and CD8 T_RM_ cells [99,100]. In the lower FRT, there are no MALT at steady state, but CD4 T_RM_ cells along with B cells, DC and macrophages form clusters during the clearance of an intravaginal HSV-2 infection. APCs including B cells and DCs facilitate CD4 T_RM_ maintenance in the lower FRT area [101,102].

The respiratory tract is an entry site for many viruses including influenza, respiratory syncytial virus (RSV) or severe acute respiratory syndrome coronavirus 2 (SARS-CoV-2). The respiratory tract can also be divided into two compartments, the upper respiratory tract (URT) and the lower respiratory tract (LRT). Nasal-associated lymphoid tissues (NALT) localized in URT contain CD4 T_RM_ cells [96]. iBALTs within the LRT are the primary niches for lung CD4 T_RM_ cells [64,103,104] following influenza infection, which is in contrast to those influenza-specific CD8 T_RM_ cells that are primarily localized to the site of regeneration in the lung parenchyma following tissue injury [33,105].

## 4. CD4 Help and Memory B and CD8 T Cell Responses

### 4.1. CD4 Help and Memory B Cell Generation

CXCR5 and PD-1 expressing CD4 helper T cells that localize in the GC are termed T_FH_ cells [106]. T_FH_ cells express the transcription factor BCL6, the cytokine IL-21 and provide CD4 T cell help to B cells in the GC. Therefore, T_FH_ cells are important in the development of long-term humoral immunity mediated by MBCs and plasma cells (PCs), which are mainly derived from GC B cells. T_FH_ cell formation goes through two sequential steps [58], including T cell priming first at the T cell zone by DCs followed by subsequent maturation in the B cell zone through interactions with B cells via ICOS-ICOS-L and MHC II-TCR. Cytokines including IL-6, IL-12 and IL-21 can promote T_FH_ formation, while IL-2 and type I IFNs potently suppress T_FH_ cell generation [58,107,108,109,110].

BCL6^lo^CD69^hi^ GC-B cells that express high levels of IRF4 favor the differentiation into PCs [111], while CCR6 has been reported as a marker for memory B cell precursors [112]. Recently, IL-9 producing T_FH_ cells have been reported to support the development of GC-derived memory precursor B cells and subsequent optimal formation of memory B cells [113]. Interestingly, the strength of the interaction between GC B and T_FH_ cells affects the formation of memory B cells. Cells prone to enter the memory B cell pool typically exhibit lower B cell receptor affinity and express high levels of Bach2, which has been found to be inversely correlated with the strength of help provided by T_FH_ cells [114].

### 4.2. CD4 T Cell Help and CD8 Memory T Cell Responses

CD4 T cell help plays an indispensable role in the primary CD8 T cell response in certain infection and/or immunization models [115,116,117]. CD4 help is critical for licensing APCs to support optimal CD8 T cell activation and differentiation [118]. In this case, interaction of CD4 T cells with APCs promotes the expression of key co-stimulatory molecules and pro-inflammatory cytokines required for maximal CD8 T cell activation [119,120]. Additionally, cytokines produced by CD4 T cells such as IL-2 can facilitate CD8 T cell expansion and effector generation [121]. However, in infectious models that generate strong inflammatory responses such as influenza infection, the primary CD8 T cell responses are largely independent of CD4 T cell help, potentially due to the direct activation of DCs by robust TLR signaling [122].

In contrast to the context-dependent roles of CD4 T cells in helping primary CD8 T cell responses, CD4 T cell help is uniformly required for the generation, maintenance and/or recall responses of memory CD8 T cell responses. To this end, CD4 T cell-derived IL-2 has been linked to promote secondary CD8 T cell responses [123,124]. Furthermore, CD4 T cells could license APCs to produce IL-15, a key cytokine involved in memory CD8 T cell formation and/or maintenance [120]. Additionally, T_REG_ cell-derived IL-10 has been shown to promote memory CD8 T cell maturation during the contraction phase via the suppression of pro-inflammatory cytokine production by DCs [125]. Lastly, activated CD4 T cells may directly interact with effector CD8 T cells via CD40-CD40L to facilitate memory CD8 T cell differentiation [126]. Regardless of the molecular cues provided by CD4 T cells for CD8 memory T cell generation, un-helped CD8 T cells are prone to apoptosis and could undergo activation-induced cell death, possibly through the induction of TRAIL expression [116,127].

Besides the role of CD4 help in the generation of circulating effector and memory T cells, recent advances have suggested that CD4 T cell help is important in mucosal T cell responses and the induction of CD8 T_RM_ responses. During HSV-2 infection, CD4 T cells control the migration of CTL through the secretion of IFN-γ and induction of local chemokine secretion in the infected tissue [128]. During influenza infection, CD4 T cell help occurs at the priming phase of T cell responses, which is critical for the development of CD8 T_RM_ cells in the lung. In the absence of CD4 T cell help, CD8 T cells failed to properly localize to the lung niches supporting optimal T_RM_ development. Furthermore, un-helped CD8 T cells exhibited high levels of T-bet expression, which interferes with CD103 expression through the modulation of TGF-β responsiveness [129]. These results demonstrate the importance of CD4 helper T cells in the formation of CD8 T_RM_ cell precursors, while the role of CD4 T cell help in CD8 T_RM_ cell maintenance has not been elucidated.

## 5. CD4 Tissue-Resident Helper Cells Coordinate T_RM_ and B_RM_ Responses in the Respiratory Mucosa

### 5.1. T_FH_-Like Cells in Non-Lymphoid Tissues

Although T_FH_ cells are generally localized in secondary lymphoid organs, T_FH_-like cells can be found in circulation or in non-lymphoid tissues [130,131]. In human joint tissue from patients with rheumatoid arthritis, a subset of T_FH_-like peripheral helper CD4 T cells (T_PH_) that exhibits potent B cell help activities was recently identified [132]. Phenotypically, T_PH_ cells share key signatures with T_FH_ cells including high levels of PD-1 expression, production of IL-21, expression of BCL6 and the lack of expression of other T helper lineage cytokines and/or transcription factors. However, T_PH_ cells have distinct features from T_FH_ cells, including the lack or low expression of CXCR5 expression and the high expression of Blimp1. T_PH_ or T_PH_-like cells have been observed in tertiary lymphoid structures developed in various inflammatory conditions such as rheumatoid arthritis (RA), Crohn’s disease and malignancy [133,134,135,136].

Tissue-infiltrating T_FH_-like cells have also been reported in animal models, particularly in inflammatory lung conditions [130,137,138]. In an HDM-induced allergic model, IL-21 producing T_FH_-like cells, which lack expression of CXCR5, were found in the inflamed lung [137]. Furthermore, IL-21 produced by those T_FH_-like cells promotes lung T_H_2 responses, eosinophil recruitment and HDM-specific IgG1 production [137]. Like human T_PH_ cells, antigen-specific T_FH_-like cells expressed high levels of IL-21, PD-1 and ICOS, but lower BCL6, CXCR5 than lymph node T_FH_ cells. In a model of LPS-adjuvanted airway immunization model, lung-infiltrating T cells were identified to exhibit follicular helper-like properties including the potential to provide help to naive B cells. These T_FH_-like cells did not express classical T_FH_ markers, CXCR5 and PD-1, but expressed molecules involved in B cell help including CD40L and IL-21. As such, these T_FH_-like cells supported the generation of GC B cells in situ within the lung [138]. Together, these data demonstrate the presence of a T_FH_-like CD4 helper population in non-lymphoid tissues, but the roles of these cells in regulating local B and CD8 memory T cells have not been examined. Furthermore, the cellular and molecular cues regulating the development of T_FH_-like cells in peripheral tissue are still unknown.

### 5.2. Identification of Tissue-Resident Helper T Cells in the Lung

As stated above, many questions remain unanswered regarding the nature and function of T_FH_-like cells in non-lymphoid tissues. First, are the T_FH_-like cells tissue-resident in non-lymphoid tissues? Second, what are the mechanisms underlying the phenotypic similarity and difference between conventional T_FH_ cells in secondary lymphoid organs and T_FH_-like cells in the non-lymphoid tissues? Third, what are the physiological functions of T_FH_-like cells in regulating local tissue immunity?

Using single-cell RNA sequencing, our group and the group of Carolyn King recently found that lung parenchyma CD4 T cells exhibit marked heterogeneity following primary influenza virus infection. In addition to traditional T_H1_-like T_RM_, T_H17_-like T_RM_ cells and tissue T_REG_ cells, lung parenchyma CD4 T cell compartment contains a T_FH_-like CD4 T cell population [64,104]. This T_FH_-like cell population appeared around two weeks after infection, following clearance of infectious virus and persisted through the memory phase, i.e., more than two months after infection. The T_FH_-like cells expressed modest BCL6 and CXCR5, and high levels of PD-1, IL-21 and FR4, thus exhibiting key T_FH_ phenotypic markers. Compared to splenic T_FH_ cells, these T_FH_-like cells had higher levels of the tissue residency gene program, the transcription factor Bhlhe40 and peripheral homing marker CXCR6, thus exhibiting T_RM_ features. Indeed, using parabiosis, we demonstrated that these tissue T_FH_-like cells were tissue-resident. Furthermore, the optimal responses of these lung T_FH_-like cells required the presence of both BCL6 and Bhlhe40, demonstrating the dual requirement of T_FH_ and T_RM_ gene programs for its development [52]. Thus, these tissue T_FH_-like cells appear to be a “hybrid” population of T_FH_ and T_RM_ cells. Based on the transcriptional, phenotypic and non-migratory characteristics, we termed these cells tissue-resident helper T cells (T_RH_).

### 5.3. Promotion of Local B Cell Immunity by T_RH_ Cells

**Figure 1:**
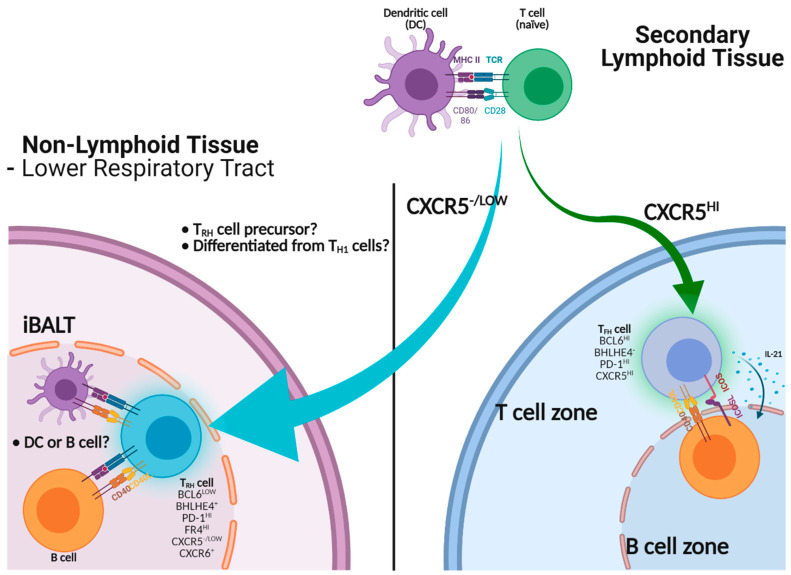
Help of B cell immunity by T_RH_ or T_FH_ cells. Activated CD4 T cells migrate into the B cell zone to become mature CXCR5^hi^ T_FH_ cells to help B cells via CD40-CD40L, ICOS-ICOS-L interactions and cytokines including IL-21. T_RH_ precursors, which express low levels of CXCR5, can infiltrate into non-lymphoid tissues such as the lung. T_RH_ precursors adapt to the lung environment to become mature T_RH_ cells, thereby assisting B cell immunity in situ through the expression of CD40L.

Like T_FH_ cells, T_RH_ cells express key B cell helping molecules including ICOS, CD40L and/or IL-21. As such, T_RH_ ablation severely impaired lung GC B cell responses and iBALT formation (Figure 1).

As discussed above, lung GC contributes to respiratory B_RM_ development and iBALT is likely a niche for lung B_RM_ cells. Consequently, we found that T_RH_ ablation impaired influenza-specific lung B_RM_ responses but not systemic B cell memory. Those B_RM_ cells that are cross-reactive to viral escape mutants were also diminished following T_RH_ depletion, suggesting that T_RH_ cells may be key for the development of broadly reactive memory B cells against heterologous influenza strains [64]. In a subsequent study, Swarnalekha et al. further showed that lung antigen-specific ASCs were significantly decreased in T_RH_ cell-ablated mice following influenza virus rechallenge [104], demonstrating the importance of T_RH_ cells in mediating memory B cell recall responses.

These studies have established the critical roles of T_RH_ in assisting the development local effector and memory B cell responses during both primary and recall responses following influenza virus infection. Swarnalekha et al. also showed that T_RH_ cells are localized within the iBALT structure, while T_H1_ T_RM_ cells are more concentrated outside or at the border of the iBALT, suggesting that T_RH_ cells may lend their help to B cells within the tertiary lymphoid organ. Similar to T_FH_ cells, T_RH_ development requires B cells and is facilitated by prolonged antigen presentation [104]. Interestingly, we found that the B cell helper function of T_RH_ cells was dependent on the CD40-CD40L interaction, but not IL-21, which is required for T_FH_-mediated B cell help. Thus, T_RH_ and T_FH_ cells may have both common and distinct B cell help mechanisms.

### 5.4. T_RH_ Cells and the Maintenance of CD8 T_RM_ Cells

During experiments dissecting the physiologic function of T_RH_ cells, perhaps the biggest surprise came from the observation that T_RH_ depletion selectively diminished a population of CD8 T_RM_ cells specific to the influenza Nucleoprotein peptide 366–374 (NP_366–374_) [64]. Previously, we found that NP_366–374_ specific CD8 T_RM_ cells receive persistent low-levels of antigenic stimulation at the memory stage, due to the delayed clearance of the NP antigen [29]. These NP_366–374_ T_RM_ cells expressed high levels of PD-1 but low levels of CD103 and possessed features of both memory and exhausted-like T cells, compared to those of conventional CD69^+^CD103^+^ T_RM_ cells [29]. Importantly, the NP_366–374_ T_RM_ cells offered critical protective function against secondary heterologous viral infection. Conversely, the blockade of PD-1 activity at the memory stage selectively expanded these T_RM_ cells and promoted the lung pathological responses [29]. Thus, those PD-1^Hi^ exhausted-like T_RM_ cells are important in maintaining the balance between T_RM_-mediated protection and pathology. When T_RH_ cells were ablated, we found that the quantity of NP_366–374_ T_RM_ cells were significantly decreased, but not those of conventional T_RM_ cells restricted to other peptides such as the influenza polymerase peptide 224–233 (PA_224–233_). Consequently, T_RM_-mediated protective immunity against heterologous viral reinfection was diminished following T_RH_ ablation. These data suggest that T_RH_ cells are vital for maintaining protective T_RM_ responses following influenza infection.

**Figure 2:**
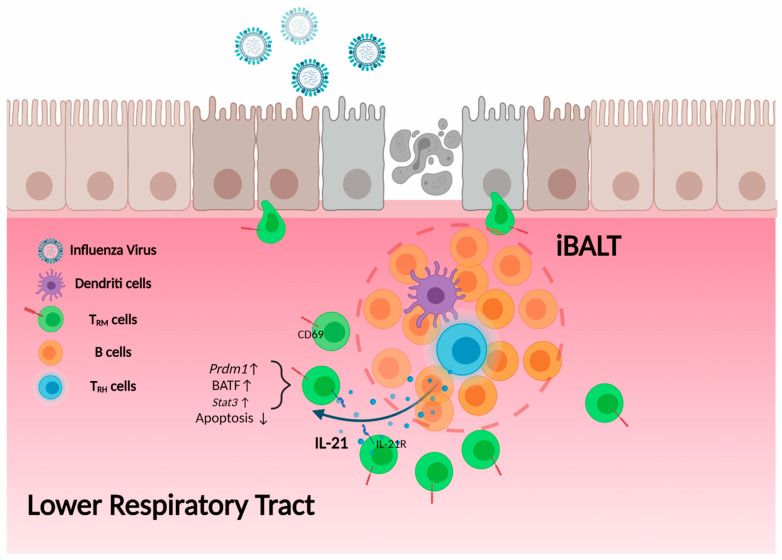
T_RH_ cell help to CD8 T_RM_ cells. T_RH_ cells, which localize within the iBALT, secrete IL-21. Influenza NP_366–374_-specific CD8 T_RM_ cells are located outside but near the border of iBALT structure and express high levels of IL-21 receptor. IL-21 secreted from T_RH_ cells promotes the expression of Blimp-1 (Prdm1), BATF and other molecules in CD8 T_RM_ cells, thereby maintaining NP_366–374_-specific T_RM_ cell retention and survival in the lung.

As discussed above, BCL6-expressing T_RH_ cells are located inside the iBALT [104]. Previous results have shown that CD8 T_RM_ cells are found outside the iBLAT (nearby border area), most of which are particularly localized near repair associated memory depots (RAMD) [105]. It is thus intriguing that T_RH_ cells facilitate NP_366–374_ T_RM_ maintenance in a contact-independent way, as T_RH_ and T_RM_ are likely localized in different lung niches. IL-21 has been showed to be a critical molecule mediating CD4 T cell help for optimal CD8 T cell responses during chronic viral infection [64]. Since NP_366–374_ T_RM_ cells exhibit features of exhausted CD8 T cells from chronic viral infection, we hypothesized that IL-21 produced by T_RH_ cells is critical to sustain NP_366–374_ T_RM_ responses (Figure 2).

Indeed, IL-21R blockade following influenza viral clearance led to diminished NP_366–374_ T_RM_ responses. Furthermore, IL-21 is mainly produced by T_RH_ cells in the lung following influenza infection [64]. These data suggest that T_RH_ cells maintain optimal T_RM_-mediated protective immunity through IL-21, which may act over a relatively longer range to provide help to CD8 T_RM_ cells compared to their help to B cells, which is mediated by cell surface molecule CD40L. Consistent with our observations, antigen-specific IL-21 producing CD4 T cells have also been identified in the brain of mice after polyomavirus (MuPyV) infection. The IL-21-producing CD4 T cells express high-affinity TCRs and T_FH_ cell markers like PD-1 and CXCR5. Importantly, in the absence of IL-21 signaling, brain CD8 T cells failed to differentiate into T_RM_ cells with sufficient CD103 expression [139]. Although it is not known whether these brain T_FH_-like cells had T_RM_ features (thus being equivalent to brain T_RH_ cells), these data do suggest that tissue T_FH_-like cells may be required for sustaining maximal CD8 T_RM_ responses in a broad spectrum of nonlymphoid tissues.

## 6. T_RH_ cells as a Potential Target for Mucosal Vaccine against Respiratory Viral Infection

Lower respiratory tract viral infections represent a major public health challenge and economic burden worldwide. In a matter of months, SARS-CoV2 infection completely altered societal norms, stagnated economies, and overwhelmed healthcare infrastructures across the globe. Annual influenza epidemics cause up to 500,000 deaths globally and there exists an ever-present threat of the emergence of a pandemic influenza strain in the future [140]. Vaccination still remains the best approach to mitigate disease burden caused by respiratory viral infection. Current vaccines against influenza and SARS-CoV2 infections are mainly administered via the systemic route, which induces strong systemic but typically weak mucosal immune responses [141,142,143,144].

The efficacy of the current influenza vaccines in providing protection against infection is still relatively limited, even in the years when the predicted vaccine strains perfectly match circulating strains. Furthermore, due to escape mutants generated by the rapid mutation of influenza virus, the current influenza vaccines require an annual update. To this end, the “holy grail” of influenza vaccine development is to create a universal influenza vaccine that can provide long-lasting and cross-reactive immunity against a broad-spectrum of influenza viral strains. It is argued that an “all-inclusive” approach, i.e., the induction of concerted immune responses, including both strong memory B and T cell responses, is needed to provide protective immunity against a wide ranges of influenza viruses [145]. Due to the nature of mucosal B_RM_ and T_RM_ responses, a mucosal vaccine that can induce strong cross-reactive B_RM_ and T_RM_ responses is more than likely a viable strategy for universal vaccine development. Since T_RH_ cells are able to provide local “help” for the development and/or maintenance of robust mucosal B and CD8 T cell responses, we argue that the promotion of T_RH_ responses by future mucosal vaccines will be key to develop successful universal influenza vaccines.

The current mRNA vaccines against SARS-CoV2 infection induce robust systemic immunity, thereby providing strong protection against viral infection and severe disease development [146]. It is still unknown whether SARS-CoV2 mRNA vaccines are able to induce effective mucosal antibody and/or B/T cell responses in humans. However, based on data generated using mouse immunization models, it seems unlikely that systemic immunization of mRNA-encoded antigens would generate strong mucosal memory T and B cells against SARS-CoV2 infection. Despite the current success of SARS-CoV2 mRNA vaccines, emerging data have suggested that the mRNA vaccine induced protective immunity may be dampened against newer variants of the SARS-CoV2 virus, particularly the delta strain [147,148]. Thus, there is still room for improvement with the development of better and broader protective SARS-CoV2 vaccines. To this end, a SARS-CoV2 vaccine capable of inducing both strong humoral and cellular (i.e., B_RM_ and/or T_RM_ development) immunity in the respiratory mucosa may ultimately meet the needs for long-lasting protection against a variety of SARS-CoV2 strains. In this case, the induction of robust respiratory T_RH_ responses again may be a pre-requirement for the success of such a vaccine.

## 7. Concluding Remarks

Memory lymphocytes establish tissue residency in the respiratory tract following pulmonary viral infection and/or mucosal vaccination. Here we reviewed the developmental cues and molecular mechanisms regulating the formation of CD4 and CD8 T_RM_ and B_RM_ cells in mucosal tissues. We put forward a T_RH_-centric model, modulating the concerted development of mucosal B and T cell memory responses following mucosal infection and immunization. We believe that the induction of a strong T_RH_ response is key to protective mucosal immunity generated by future universal vaccine candidates. Conversely, dysregulated T_RH_ responses may contribute to the development pulmonary inflammation in various disease conditions including asthma or long-term chronic sequelae following respiratory viral infections [149,150].

## Data Availability

Not applicable.

## References

[1] Sallusto F., Lenig D., Förster R., Lipp M., Lanzavecchia A. (1999). Two subsets of memory T lymphocytes with distinct homing potentials and effector functions. Nature.

[2] Farber D.L., Ahmadzadeh M. (2002). Dissecting the complexity of the memory T cell response. Immunol. Res..

[3] Mueller S.N., Gebhardt T. (2013). Carbone FR, Heath WR. Memory T cell subsets, migration patterns, and tissue residence. Annu. Rev. Immunol..

[4] Martin M.D., Badovinac V.P. (2018). Defining Memory CD8 T Cell. Front. Immunol..

[5] Gerlach C., Moseman E.A., Loughhead S.M., Alvarez D., Zwijnenburg A.J., Waanders L., Garg R., de la Torre J.C., von Andrian U.H. (2016). The Chemokine Receptor CX3CR1 Defines Three Antigen-Experienced CD8 T Cell Subsets with Distinct Roles in Immune Surveillance and Homeostasis. Immunity.

[6] Masopust D., Soerens A.G. (2019). Tissue-Resident T Cells and Other Resident Leukocytes. Annu. Rev. Immunol..

[7] Gebhardt T., Wakim L.M., Eidsmo L., Reading P.C., Heath W.R., Carbone F.R. (2009). Memory T cells in nonlymphoid tissue that provide enhanced local immunity during infection with herpes simplex virus. Nat. Immunol..

[8] Steinbach K., Vincenti I., Merkler D. (2018). Resident-Memory T Cells in Tissue-Restricted Immune Responses. For Better or Worse?. Front. Immunol..

[9] Grau-Expósito J., Sánchez-Gaona N., Massana N., Suppi M., Astorga-Gamaza A., Perea D., Rosado J., Falcó A., Kirkegaard C., Torrella A. (2021). Peripheral and lung resident memory T cell responses against SARS-CoV-2. Nat. Commun..

[10] Mackay L.K., Minnich M., Kragten N.A., Liao Y., Nota B., Seillet C., Zaid A., Man K., Preston S., Freestone D. (2016). Hobit and Blimp1 instruct a universal transcriptional program of tissue residency in lymphocytes. Science.

[11] Blanchard-Rohner G., Pulickal A.S., Jol-van der Zijde C.M., Snape M.D., Pollard A.J. (2009). Appearance of peripheral blood plasma cells and memory B cells in a primary and secondary immune response in humans. Blood.

[12] De Silva N.S., Klein U. (2015). Dynamics of B cells in germinal centres. Nat. Rev. Immunol..

[13] Liu D., Xu H., Shih C., Wan Z., Ma X., Ma W., Luo D., Qi H. (2015). T-B-cell entanglement and ICOSL-driven feed-forward regulation of germinal centre reaction. Nature.

[14] Han S., Hathcock K., Zheng B., Kepler T.B., Hodes R., Kelsoe G. (1995). Cellular interaction in germinal centers. Roles of CD40 ligand and B7-2 in established germinal centers. J. Immunol..

[15] Zuccarino-Catania G.V., Sadanand S., Weisel F.J., Tomayko M.M., Meng H., Kleinstein S.H., Good-Jacobson K.L., Shlomchik M.J. (2014). CD80 and PD-L2 define functionally distinct memory B cell subsets that are independent of antibody isotype. Nat. Immunol..

[16] Dugan H.L., Guthmiller J.J., Arevalo P., Huang M., Chen Y.Q., Neu K.E., Henry C., Zheng N.Y., Lan L.Y., Tepora M.E. (2020). Preexisting immunity shapes distinct antibody landscapes after influenza virus infection and vaccination in humans. Sci. Transl. Med..

[17] Mesin L., Schiepers A., Ersching J., Barbulescu A., Cavazzoni C.B., Angelini A., Okada T., Kurosaki T., Victora G.D. (2020). Restricted Clonality and Limited Germinal Center Reentry Characterize Memory B Cell Reactivation by Boosting. Cell.

[18] Allie S.R., Bradley J.E., Mudunuru U., Schultz M.D., Graf B.A., Lund F.E., Randall T.D. (2019). The establishment of resident memory B cells in the lung requires local antigen encounter. Nat. Immunol..

[19] Mani V., Bromley S.K., Äijö T., Mora-Buch R., Carrizosa E., Warner R.D., Hamze M., Sen D.R., Chasse A.Y., Lorant A. (2019). Migratory DCs activate TGF-β to precondition naïve CD8(+) T cells for tissue-resident memory fate. Science.

[20] Kim T.S., Gorski S.A., Hahn S., Murphy K.M., Braciale T.J. (2014). Distinct dendritic cell subsets dictate the fate decision between effector and memory CD8(+) T cell differentiation by a CD24-dependent mechanism. Immunity.

[21] Kok L., Dijkgraaf F.E., Urbanus J., Bresser K., Vredevoogd D.W., Cardoso R.F., Perié L., Beltman J.B., Schumacher T.N. (2020). A committed tissue-resident memory T cell precursor within the circulating CD8+ effector T cell pool. J. Exp. Med..

[22] Casey K.A., Fraser K.A., Schenkel J.M., Moran A., Abt M.C., Beura L.K., Lucas P.J., Artis D., Wherry E.J., Hogquist K. (2012). Antigen-independent differentiation and maintenance of effector-like resident memory T cells in tissues. J. Immunol..

[23] Mackay L.K., Stock A.T., Ma J.Z., Jones C.M., Kent S.J., Mueller S.N., Heath W.R., Carbone F.R., Gebhardt T. (2012). Long-lived epithelial immunity by tissue-resident memory T (TRM) cells in the absence of persisting local antigen presentation. Proc. Natl. Acad. Sci. USA.

[24] Holz L.E., Prier J.E., Freestone D., Steiner T.M., English K., Johnson D.N., Mollard V., Cozijnsen A., Davey G.M., Godfrey D.I. (2018). CD8(+) T Cell Activation Leads to Constitutive Formation of Liver Tissue-Resident Memory T Cells that Seed a Large and Flexible Niche in the Liver. Cell Rep..

[25] Khan T.N., Mooster J.L., Kilgore A.M., Osborn J.F., Nolz J.C. (2016). Local antigen in nonlymphoid tissue promotes resident memory CD8+ T cell formation during viral infection. J. Exp. Med..

[26] Fernandez-Ruiz D., Ng W.Y., Holz L.E., Ma J.Z., Zaid A., Wong Y.C., Lau L.S., Mollard V., Cozijnsen A., Collins N. (2016). Liver-Resident Memory CD8(+) T Cells Form a Front-Line Defense against Malaria Liver-Stage Infection. Immunity.

[27] Wakim L.M., Woodward-Davis A., Bevan M.J. (2010). Memory T cells persisting within the brain after local infection show functional adaptations to their tissue of residence. Proc. Natl. Acad. Sci. USA.

[28] Wijeyesinghe S., Beura L.K., Pierson M.J., Stolley J.M., Adam O.A., Ruscher R., Steinert E.M., Rosato P.C., Vezys V., Masopust D. (2021). Expansible residence decentralizes immune homeostasis. Nature.

[29] Wang Z., Wang S., Goplen N.P., Li C., Cheon I.S., Dai Q., Huang S., Shan J., Ma C., Ye Z. (2019). PD-1(hi) CD8(+) resident memory T cells balance immunity and fibrotic sequelae. Sci. Immunol..

[30] Uddbäck I., Cartwright E.K., Schøller A.S., Wein A.N., Hayward S.L., Lobby J., Takamura S., Thomsen A.R., Kohlmeier J.E., Christensen J.P. (2021). Long-term maintenance of lung resident memory T cells is mediated by persistent antigen. Mucosal Immunol..

[31] Mackay L.K., Braun A., Macleod B.L., Collins N., Tebartz C., Bedoui S., Carbone F.R., Gebhardt T. (2015). Cutting edge. CD69 interference with sphingosine-1-phosphate receptor function regulates peripheral T cell retention. J. Immunol..

[32] Shiow L.R., Rosen D.B., Brdicková N., Xu Y., An J., Lanier L.L., Cyster J.G., Matloubian M. (2006). CD69 acts downstream of interferon-alpha/beta to inhibit S1P1 and lymphocyte egress from lymphoid organs. Nature.

[33] Takamura S., Yagi H., Hakata Y., Motozono C., McMaster S.R., Masumoto T., Fujisawa M., Chikaishi T., Komeda J., Itoh J. (2016). Specific niches for lung-resident memory CD8+ T cells at the site of tissue regeneration enable CD69-independent maintenance. J. Exp. Med..

[34] Carlson C.M., Endrizzi B.T., Wu J., Ding X., Weinreich M.A., Walsh E.R., Wani M.A., Lingrel J.B., Hogquist K.A., Jameson S.C. (2006). Kruppel-like factor 2 regulates thymocyte and T-cell migration. Nature.

[35] Lee Y.T., Suarez-Ramirez J.E., Wu T., Redman J.M., Bouchard K., Hadley G.A., Cauley L.S. (2011). Environmental and antigen receptor-derived signals support sustained surveillance of the lungs by pathogen-specific cytotoxic T lymphocytes. J. Virol..

[36] Thom J.T., Weber T.C., Walton S.M., Torti N., Oxenius A. (2015). The Salivary Gland Acts as a Sink for Tissue-Resident Memory CD8(+) T Cells, Facilitating Protection from Local Cytomegalovirus Infection. Cell Rep..

[37] Mackay L.K., Rahimpour A., Ma J.Z., Collins N., Stock A.T., Hafon M.L., Vega-Ramos J., Lauzurica P., Mueller S.N., Stefanovic T. (2013). The developmental pathway for CD103(+)CD8+ tissue-resident memory T cells of skin. Nat. Immunol..

[38] Sheridan B.S., Pham Q.M., Lee Y.T., Cauley L.S., Puddington L., Lefrançois L. (2014). Oral infection drives a distinct population of intestinal resident memory CD8(+) T cells with enhanced protective function. Immunity.

[39] Schluns K.S., Williams K., Ma A., Zheng X.X., Lefrançois L. (2002). Cutting edge: Requirement for IL-15 in the generation of primary and memory antigen-specific CD8 T cells. J. Immunol..

[40] Sowell R.T., Goldufsky J.W., Rogozinska M., Quiles Z., Cao Y., Castillo E.F., Finnegan A., Marzo A.L. (2017). IL-15 Complexes Induce Migration of Resting Memory CD8 T Cells into Mucosal Tissues. J. Immunol..

[41] Burchill M.A., Goetz C.A., Prlic M., O’Neil J.J., Harmon I.R., Bensinger S.J., Turka L.A., Brennan P., Jameson S.C., Farrar M.A. (2003). Distinct effects of STAT5 activation on CD4+ and CD8+ T cell homeostasis: Development of CD4+CD25+ regulatory T cells versus CD8+ memory T cells. J. Immunol..

[42] Hand T.W., Cui W., Jung Y.W., Sefik E., Joshi N.S., Chandele A., Liu Y., Kaech S.M. (2010). Differential effects of STAT5 and PI3K/AKT signaling on effector and memory CD8 T-cell survival. Proc. Natl. Acad. Sci. USA.

[43] Adachi T., Kobayashi T., Sugihara E., Yamada T., Ikuta K., Pittaluga S., Saya H., Amagai M., Nagao K. (2015). Hair follicle-derived IL-7 and IL-15 mediate skin-resident memory T cell homeostasis and lymphoma. Nat. Med..

[44] Mackay L.K., Wynne-Jones E., Freestone D., Pellicci D.G., Mielke L.A., Newman D.M., Braun A., Masson F., Kallies A., Belz G.T. (2015). T-box Transcription Factors Combine with the Cytokines TGF-beta and IL-15 to Control Tissue-Resident Memory T Cell Fate. Immunity.

[45] Skon C.N., Lee J.Y., Anderson K.G., Masopust D., Hogquist K.A., Jameson S.C. (2013). Transcriptional downregulation of S1pr1 is required for the establishment of resident memory CD8+ T cells. Nat. Immunol..

[46] Bergsbaken T., Bevan M.J., Fink P.J. (2017). Local Inflammatory Cues Regulate Differentiation and Persistence of CD8(+) Tissue-Resident Memory T Cells. Cell Rep..

[47] Grueter B., Petter M., Egawa T., Laule-Kilian K., Aldrian C.J., Wuerch A., Ludwig Y., Fukuyama H., Wardemann H., Waldschuetz R. (2005). Runx3 regulates integrin alpha E/CD103 and CD4 expression during development of CD4-/CD8+ T cells. J. Immunol..

[48] Wang D., Diao H., Getzler A.J., Rogal W., Frederick M.A., Milner J., Yu B., Crotty S., Goldrath A.W., Pipkin M.E. (2018). The Transcription Factor Runx3 Establishes Chromatin Accessibility of cis-Regulatory Landscapes that Drive Memory Cytotoxic T Lymphocyte Formation. Immunity.

[49] Hombrink P., Helbig C., Backer R.A., Piet B., Oja A.E., Stark R., Brasser G., Jongejan A., Jonkers R.E., Nota B. (2016). Programs for the persistence, vigilance and control of human CD8(+) lung-resident memory T cells. Nat. Immunol..

[50] Elyaman W., Bassil R., Bradshaw E.M., Orent W., Lahoud Y., Zhu B., Radtke F., Yagita H., Khoury S.J. (2012). Notch receptors and Smad3 signaling cooperate in the induction of interleukin-9-producing T cells. Immunity.

[51] Blokzijl A., Dahlqvist C., Reissmann E., Falk A., Moliner A., Lendahl U., Ibáñez C.F. (2003). Cross-talk between the Notch and TGF-beta signaling pathways mediated by interaction of the Notch intracellular domain with Smad3. J. Cell Biol..

[52] Li C., Zhu B., Son Y.M., Wang Z., Jiang L., Xiang M., Ye Z., Beckermann K.E., Wu Y., Jenkins J.W. (2019). The Transcription Factor Bhlhe40 Programs Mitochondrial Regulation of Resident CD8(+) T Cell Fitness and Functionality. Immunity.

[53] Zaid A., Mackay L.K., Rahimpour A., Braun A., Veldhoen M., Carbone F.R., Manton J.H., Heath W.R., Mueller S.N. (2014). Persistence of skin-resident memory T cells within an epidermal niche. Proc. Natl. Acad. Sci. USA.

[54] Boddupalli C.S., Nair S., Gray S.M., Nowyhed H.N., Verma R., Gibson J.A., Abraham C., Narayan D., Vasquez J., Hedrick C.C. (2016). ABC transporters and NR4A1 identify a quiescent subset of tissue-resident memory T cells. J. Clin. Investig..

[55] Halliley J.L., Tipton C.M., Liesveld J., Rosenberg A.F., Darce J., Gregoretti I.V., Popova L., Kaminiski D., Fucile C.F., Albizua I. (2015). Long-Lived Plasma Cells Are Contained within the CD19(-)CD38(hi)CD138(+) Subset in Human Bone Marrow. Immunity.

[56] Tarlinton D., Good-Jacobson K. (2013). Diversity among memory B cells: Origin, consequences, and utility. Science.

[57] Akkaya M., Kwak K., Pierce S.K. (2020). B cell memory: Building two walls of protection against pathogens. Nat. Rev. Immunol..

[58] Crotty S. (2019). T Follicular Helper Cell Biology. A Decade of Discovery and Diseases. Immunity.

[59] Onodera T., Takahashi Y., Yokoi Y., Ato M., Kodama Y., Hachimura S., Kurosaki T., Kobayashi K. (2012). Memory B cells in the lung participate in protective humoral immune responses to pulmonary influenza virus reinfection. Proc. Natl. Acad. Sci. USA.

[60] Weisel N.M., Weisel F.J., Farber D.L., Borghesi L.A., Shen Y., Ma W., Luning Prak E.T., Shlomchik M.J. (2020). Comprehensive analyses of B-cell compartments across the human body reveal novel subsets and a gut-resident memory phenotype. Blood.

[61] Boyden A.W., Legge K.L., Waldschmidt T.J. (2012). Pulmonary infection with influenza A virus induces site-specific germinal center and T follicular helper cell responses. PLoS ONE.

[62] Allie S.R., Randall T.D. (2017). Pulmonary immunity to viruses. Clin. Sci..

[63] Moyron-Quiroz J.E., Rangel-Moreno J., Hartson L., Kusser K., Tighe M.P., Klonowski K.D., Lefrançois L., Cauley L.S., Harmsen A.G., Lund F.E. (2006). Persistence and responsiveness of immunologic memory in the absence of secondary lymphoid organs. Immunity.

[64] Son Y.M., Cheon I.S., Wu Y., Li C., Wang Z., Gao X., Chen Y., Takahashi Y., Fu Y.X., Dent A.L. (2021). Tissue-resident CD4(+) T helper cells assist the development of protective respiratory B and CD8(+) T cell memory responses. Sci. Immunol..

[65] Adachi Y., Onodera T., Yamada Y., Daio R., Tsuiji M., Inoue T., Kobayashi K., Kurosaki T., Ato M., Takahashi Y. (2015). Distinct germinal center selection at local sites shapes memory B cell response to viral escape. J. Exp. Med..

[66] Allie S.R., Randall T.D. (2020). Resident Memory B Cells. Viral Immunol..

[67] Dogan I., Bertocci B., Vilmont V., Delbos F., Mégret J., Storck S., Reynaud C.A., Weill J.C. (2009). Multiple layers of B cell memory with different effector functions. Nat. Immunol..

[68] McHeyzer-Williams L.J., Milpied P.J., Okitsu S.L., McHeyzer-Williams M.G. (2015). Class-switched memory B cells remodel BCRs within secondary germinal centers. Nat. Immunol..

[69] Pape K.A., Taylor J.J., Maul R.W., Gearhart P.J., Jenkins M.K. (2011). Different B cell populations mediate early and late memory during an endogenous immune response. Science.

[70] Tarlinton D. (1997). Antigen presentation by memory B cells: The sting is in the tail. Science.

[71] Barker K.A., Etesami N.S., Shenoy A.T., Arafa E.I., Lyon de Ana C., Smith N.M., Martin I.M., Goltry W.N., Barron A.M., Browning J.L. (2021). Lung-resident memory B cells protect against bacterial pneumonia. J. Clin. Investig..

[72] Beura L.K., Fares-Frederickson N.J., Steinert E.M., Scott M.C., Thompson E.A., Fraser K.A., Schenkel J.M., Vezys V., Masopust D. (2019). CD4(+) resident memory T cells dominate immunosurveillance and orchestrate local recall responses. J. Exp. Med..

[73] Kumar B.V., Ma W., Miron M., Granot T., Guyer R.S., Carpenter D.J., Senda T., Sun X., Ho S.H., Lerner H. (2017). Human Tissue-Resident Memory T Cells Are Defined by Core Transcriptional and Functional Signatures in Lymphoid and Mucosal Sites. Cell Rep..

[74] Oja A.E., Piet B., Helbig C., Stark R., van der Zwan D., Blaauwgeers H., Remmerswaal E.B.M., Amsen D., Jonkers R.E., Moerland P.D. (2018). Trigger-happy resident memory CD4(+) T cells inhabit the human lungs. Mucosal Immunol..

[75] Schreiner D., King C.G. (2018). CD4+ Memory T Cells at Home in the Tissue: Mechanisms for Health and Disease. Front. Immunol..

[76] Pruner K.B., Pepper M. (2021). Local memory CD4 T cell niches in respiratory viral infection. J. Exp. Med..

[77] Turner D.L., Bickham K.L., Thome J.J., Kim C.Y., D’Ovidio F., Wherry E.J., Farber D.L. (2014). Lung niches for the generation and maintenance of tissue-resident memory T cells. Mucosal Immunol..

[78] Teijaro J.R., Turner D., Pham Q., Wherry E.J., Lefrançois L., Farber D.L. (2011). Cutting edge: Tissue-retentive lung memory CD4 T cells mediate optimal protection to respiratory virus infection. J. Immunol..

[79] Glennie N.D., Volk S.W., Scott P. (2017). Skin-resident CD4+ T cells protect against Leishmania major by recruiting and activating inflammatory monocytes. PLoS Pathog..

[80] Glennie N.D., Yeramilli V.A., Beiting D.P., Volk S.W., Weaver C.T., Scott P. (2015). Skin-resident memory CD4+ T cells enhance protection against Leishmania major infection. J. Exp. Med..

[81] Romagnoli P.A., Fu H.H., Qiu Z., Khairallah C., Pham Q.M., Puddington L., Khanna K.M., Lefrançois L., Sheridan B.S. (2017). Differentiation of distinct long-lived memory CD4 T cells in intestinal tissues after oral Listeria monocytogenes infection. Mucosal Immunol..

[82] Sakai S., Kauffman K.D., Schenkel J.M., McBerry C.C., Mayer-Barber K.D., Masopust D., Barber D.L. (2014). Cutting edge: Control of Mycobacterium tuberculosis infection by a subset of lung parenchyma-homing CD4 T cells. J. Immunol..

[83] Sallin M.A., Sakai S., Kauffman K.D., Young H.A., Zhu J., Barber D.L. (2017). Th1 Differentiation Drives the Accumulation of Intravascular, Non-protective CD4 T Cells during Tuberculosis. Cell Rep..

[84] Chapman T.J., Topham D.J. (2010). Identification of a unique population of tissue-memory CD4+ T cells in the airways after influenza infection that is dependent on the integrin VLA-1. J. Immunol..

[85] Rahimi R.A., Nepal K., Cetinbas M., Sadreyev R.I., Luster A.D. (2020). Distinct functions of tissue-resident and circulating memory Th2 cells in allergic airway disease. J. Exp. Med..

[86] Steinfelder S., Rausch S., Michael D., Kühl A.A., Hartmann S. (2017). Intestinal helminth infection induces highly functional resident memory CD4(+) T cells in mice. Eur. J. Immunol..

[87] Park C.O., Fu X., Jiang X., Pan Y., Teague J.E., Collins N., Tian T., O’Malley J.T., Emerson R.O., Kim J.H. (2018). Staged development of long-lived T-cell receptor alphabeta TH17 resident memory T-cell population to Candida albicans after skin infection. J. Allergy Clin. Immunol..

[88] Ogongo P., Tezera L.B., Ardain A., Nhamoyebonde S., Ramsuran D., Singh A., Ng’oepe A., Karim F., Naidoo T., Khan K. (2021). Tissue-resident-like CD4+ T cells secreting IL-17 control Mycobacterium tuberculosis in the human lung. J. Clin. Investig..

[89] Ichikawa T., Hirahara K., Kokubo K., Kiuchi M., Aoki A., Morimoto Y., Kumagai J., Onodera A., Mato N., Tumes D.J. (2019). CD103(hi) T(reg) cells constrain lung fibrosis induced by CD103(lo) tissue-resident pathogenic CD4 T cells. Nat. Immunol..

[90] Arpaia N., Green J.A., Moltedo B., Arvey A., Hemmers S., Yuan S., Treuting P.M., Rudensky A.Y. (2015). A Distinct Function of Regulatory T Cells in Tissue Protection. Cell.

[91] McKinstry K.K., Strutt T.M., Bautista B., Zhang W., Kuang Y., Cooper A.M., Swain S.L. (2014). Effector CD4 T-cell transition to memory requires late cognate interactions that induce autocrine IL-2. Nat. Commun..

[92] Hondowicz B.D., An D., Schenkel J.M., Kim K.S., Steach H.R., Krishnamurty A.T., Keitany G.J., Garza E.N., Fraser K.A., Moon J.J. (2016). Interleukin-2-Dependent Allergen-Specific Tissue-Resident Memory Cells Drive Asthma. Immunity.

[93] Hondowicz B.D., Kim K.S., Ruterbusch M.J., Keitany G.J., Pepper M. (2018). IL-2 is required for the generation of viral-specific CD4(+) Th1 tissue-resident memory cells and B cells are essential for maintenance in the lung. Eur. J. Immunol..

[94] Kang M.C., Choi D.H., Choi Y.W., Park S.J., Namkoong H., Park K.S., Ahn S.S., Surh C.D., Yoon S.W., Kim D.J. (2015). Intranasal Introduction of Fc-Fused Interleukin-7 Provides Long-Lasting Prophylaxis against Lethal Influenza Virus Infection. J. Virol..

[95] Strutt T.M., Dhume K., Finn C.M., Hwang J.H., Castonguay C., Swain S.L., McKinstry K.K. (2018). IL-15 supports the generation of protective lung-resident memory CD4 T cells. Mucosal Immunol..

[96] Takamura S. (2018). Niches for the Long-Term Maintenance of Tissue-Resident Memory T Cells. Front. Immunol..

[97] Collins N., Jiang X., Zaid A., Macleod B.L., Li J., Park C.O., Haque A., Bedoui S., Heath W.R., Mueller S.N. (2016). Skin CD4(+) memory T cells exhibit combined cluster-mediated retention and equilibration with the circulation. Nat. Commun..

[98] Gebhardt T., Whitney P.G., Zaid A., Mackay L.K., Brooks A.G., Heath W.R., Carbone F.R., Mueller S.N. (2011). Different patterns of peripheral migration by memory CD4+ and CD8+ T cells. Nature.

[99] Gondek D.C., Olive A.J., Stary G., Starnbach M.N. (2012). CD4+ T cells are necessary and sufficient to confer protection against Chlamydia trachomatis infection in the murine upper genital tract. J. Immunol..

[100] Johnson R.M., Brunham R.C. (2016). Tissue-Resident T Cells as the Central Paradigm of Chlamydia Immunity. Infect. Immun..

[101] Johnson R.M., Yu H., Strank N.O., Karunakaran K., Zhu Y., Brunham R.C. (2018). B Cell Presentation of Chlamydia Antigen Selects Out Protective CD4γ13 T Cells: Implications for Genital Tract Tissue-Resident Memory Lymphocyte Clusters. Infect. Immun..

[102] Iijima N., Linehan M.M., Zamora M., Butkus D., Dunn R., Kehry M.R., Laufer T.M., Iwasaki A. (2008). Dendritic cells and B cells maximize mucosal Th1 memory response to herpes simplex virus. J. Exp. Med..

[103] Hwang J.Y., Randall T.D., Silva-Sanchez A. (2016). Inducible Bronchus-Associated Lymphoid Tissue: Taming Inflammation in the Lung. Front. Immunol..

[104] Swarnalekha N., Schreiner D., Litzler L.C., Iftikhar S., Kirchmeier D., Kunzli M., Son Y.M., Sun J., Moreira E.A., King C.G. (2021). T resident helper cells promote humoral responses in the lung. Sci. Immunol..

[105] Yoshizawa A., Bi K., Keskin D.B., Zhang G., Reinhold B., Reinherz E.L. (2018). TCR-pMHC encounter differentially regulates transcriptomes of tissue-resident CD8 T cells. Eur. J. Immunol..

[106] Crotty S. (2011). Follicular helper CD4 T cells (TFH). Annu. Rev. Immunol..

[107] Schmitt N., Bustamante J., Bourdery L., Bentebibel S.E., Boisson-Dupuis S., Hamlin F., Tran M.V., Blankenship D., Pascual V., Savino D.A. (2013). IL-12 receptor β1 deficiency alters in vivo T follicular helper cell response in humans. Blood.

[108] Johnston R.J., Choi Y.S., Diamond J.A., Yang J.A., Crotty S. (2012). STAT5 is a potent negative regulator of TFH cell differentiation. J. Exp. Med..

[109] Ballesteros-Tato A., Leon B., Graf B.A., Moquin A., Adams P.S., Lund F.E., Randall T.D. (2012). Interleukin-2 inhibits germinal center formation by limiting T follicular helper cell differentiation. Immunity.

[110] DiToro D., Winstead C.J., Pham D., Witte S., Andargachew R., Singer J.R., Wilson C.G., Zindl C.L., Luther R.J., Silberger D.J. (2018). Differential IL-2 expression defines developmental fates of follicular versus nonfollicular helper T cells. Science.

[111] Ise W., Fujii K., Shiroguchi K., Ito A., Kometani K., Takeda K., Kawakami E., Yamashita K., Suzuki K., Okada T. (2018). T Follicular Helper Cell-Germinal Center B Cell Interaction Strength Regulates Entry into Plasma Cell or Recycling Germinal Center Cell Fate. Immunity.

[112] Suan D., Kräutler N.J., Maag J.L.V., Butt D., Bourne K., Hermes J.R., Avery D.T., Young C., Statham A., Elliott M. (2017). CCR6 Defines Memory B Cell Precursors in Mouse and Human Germinal Centers, Revealing Light-Zone Location and Predominant Low Antigen Affinity. Immunity.

[113] Wang Y., Shi J., Yan J., Xiao Z., Hou X., Lu P., Hou S., Mao T., Liu W., Ma Y. (2017). Germinal-center development of memory B cells driven by IL-9 from follicular helper T cells. Nat. Immunol..

[114] Shinnakasu R., Inoue T., Kometani K., Moriyama S., Adachi Y., Nakayama M., Takahashi Y., Fukuyama H., Okada T., Kurosaki T. (2016). Regulated selection of germinal-center cells into the memory B cell compartment. Nat. Immunol..

[115] Phares T.W., Stohlman S.A., Hwang M., Min B., Hinton D.R., Bergmann C.C. (2012). CD4 T cells promote CD8 T cell immunity at the priming and effector site during viral encephalitis. J. Virol..

[116] Janssen E.M., Droin N.M., Lemmens E.E., Pinkoski M.J., Bensinger S.J., Ehst B.D., Griffith T.S., Green D.R., Schoenberger S.P. (2005). CD4+ T-cell help controls CD8+ T-cell memory via TRAIL-mediated activation-induced cell death. Nature.

[117] Bevan M.J. (2004). Helping the CD8(+) T-cell response. Nat. Rev. Immunol..

[118] Smith C.M., Wilson N.S., Waithman J., Villadangos J.A., Carbone F.R., Heath W.R., Belz G.T. (2004). Cognate CD4(+) T cell licensing of dendritic cells in CD8(+) T cell immunity. Nat. Immunol..

[119] Swain S.L., McKinstry K.K., Strutt T.M. (2012). Expanding roles for CD4^+^ T cells in immunity to viruses. Nat. Rev. Immunol..

[120] Oh S., Perera L.P., Terabe M., Ni L., Waldmann T.A., Berzofsky J.A. (2008). IL-15 as a mediator of CD4+ help for CD8+ T cell longevity and avoidance of TRAIL-mediated apoptosis. Proc. Natl. Acad. Sci. USA.

[121] Williams M.A., Tyznik A.J., Bevan M.J. (2006). Interleukin-2 signals during priming are required for secondary expansion of CD8+ memory T cells. Nature.

[122] Johnson S., Zhan Y., Sutherland R.M., Mount A.M., Bedoui S., Brady J.L., Carrington E.M., Brown L.E., Belz G.T., Heath W.R. (2009). Selected Toll-like receptor ligands and viruses promote helper-independent cytotoxic T cell priming by upregulating CD40L on dendritic cells. Immunity.

[123] Mitchell D.M., Ravkov E.V., Williams M.A. (2010). Distinct roles for IL-2 and IL-15 in the differentiation and survival of CD8+ effector and memory T cells. J. Immunol..

[124] Kalia V., Sarkar S. (2018). Regulation of Effector and Memory CD8 T Cell Differentiation by IL-2-A Balancing Act. Front. Immunol..

[125] Laidlaw B.J., Cui W., Amezquita R.A., Gray S.M., Guan T., Lu Y., Kobayashi Y., Flavell R.A., Kleinstein S.H., Craft J. (2015). Production of IL-10 by CD4(+) regulatory T cells during the resolution of infection promotes the maturation of memory CD8(+) T cells. Nat. Immunol..

[126] Bourgeois C., Rocha B., Tanchot C. (2002). A role for CD40 expression on CD8+ T cells in the generation of CD8+ T cell memory. Science.

[127] Badovinac V.P., Messingham K.A., Griffith T.S., Harty J.T. (2006). TRAIL deficiency delays, but does not prevent, erosion in the quality of “helpless” memory CD8 T cells. J. Immunol..

[128] Nakanishi Y., Lu B., Gerard C., Iwasaki A. (2009). CD8(+) T lymphocyte mobilization to virus-infected tissue requires CD4(+) T-cell help. Nature.

[129] Laidlaw B.J., Zhang N., Marshall H.D., Staron M.M., Guan T., Hu Y., Cauley L.S., Craft J., Kaech S.M. (2014). CD4+ T cell help guides formation of CD103+ lung-resident memory CD8+ T cells during influenza viral infection. Immunity.

[130] Hutloff A. (2018). T Follicular Helper-Like Cells in Inflamed Non-Lymphoid Tissues. Front. Immunol..

[131] Yoshitomi H., Ueno H. (2021). Shared and distinct roles of T peripheral helper and T follicular helper cells in human diseases. Cell Mol. Immunol..

[132] Rao D.A., Gurish M.F., Marshall J.L., Slowikowski K., Fonseka C.Y., Liu Y., Donlin L.T., Henderson L.A., Wei K., Mizoguchi F. (2017). Pathologically expanded peripheral T helper cell subset drives B cells in rheumatoid arthritis. Nature.

[133] Manzo A., Vitolo B., Humby F., Caporali R., Jarrossay D., Dell’accio F., Ciardelli L., Uguccioni M., Montecucco C., Pitzalis C. (2008). Mature antigen-experienced T helper cells synthesize and secrete the B cell chemoattractant CXCL13 in the inflammatory environment of the rheumatoid joint. Arthritis Rheum..

[134] Kobayashi S., Murata K., Shibuya H., Morita M., Ishikawa M., Furu M., Ito H., Ito J., Matsuda S., Watanabe T. (2013). A distinct human CD4+ T cell subset that secretes CXCL13 in rheumatoid synovium. Arthritis Rheum..

[135] Rubin S.J.S., Bai L., Haileselassie Y., Garay G., Yun C., Becker L., Streett S.E., Sinha S.R., Habtezion A. (2019). Mass cytometry reveals systemic and local immune signatures that distinguish inflammatory bowel diseases. Nat. Commun..

[136] Pitzalis C., Jones G.W., Bombardieri M., Jones S.A. (2014). Ectopic lymphoid-like structures in infection, cancer and autoimmunity. Nat. Rev. Immunol..

[137] Coquet J.M., Schuijs M.J., Smyth M.J., Deswarte K., Beyaert R., Braun H., Boon L., Karlsson Hedestam G.B., Nutt S.L., Hammad H. (2015). Interleukin-21-Producing CD4(+) T Cells Promote Type 2 Immunity to House Dust Mites. Immunity.

[138] Vu Van D., Beier K.C., Pietzke L.J., Al Baz M.S., Feist R.K., Gurka S., Hamelmann E., Kroczek R.A., Hutloff A. (2016). Local T/B cooperation in inflamed tissues is supported by T follicular helper-like cells. Nat. Commun..

[139] Ren H.M., Kolawole E.M., Ren M., Jin G., Netherby-Winslow C.S., Wade Q., Shwetank Rahman Z.S.M., Evavold B.D., Lukacher A.E. (2020). IL-21 from high-affinity CD4 T cells drives differentiation of brain-resident CD8 T cells during persistent viral infection. Sci. Immunol..

[140] Flerlage T., Boyd D.F., Meliopoulos V., Thomas P.G., Schultz-Cherry S. (2021). Influenza virus and SARS-CoV-2. pathogenesis and host responses in the respiratory tract. Nat. Rev. Microbiol..

[141] Sano K., Ainai A., Suzuki T., Hasegawa H. (2017). The road to a more effective influenza vaccine: Up to date studies and future prospects. Vaccine.

[142] Hellfritzsch M., Scherließ R. (2019). Mucosal Vaccination via the Respiratory Tract. Pharmaceutics.

[143] Pilkington E.H., Suys E.J.A., Trevaskis N.L., Wheatley A.K., Zukancic D., Algarni A., Al-Wassiti H., Davis T.P., Pouton C.W., Kent S.J. (2021). From influenza to COVID-19: Lipid nanoparticle mRNA vaccines at the frontiers of infectious diseases. Acta Biomater..

[144] Wang R., Liu M., Liu J. (2021). The Association between Influenza Vaccination and COVID-19 and Its Outcomes: A Systematic Review and Meta-Analysis of Observational Studies. Vaccines.

[145] Estrada L.D., Schultz-Cherry S. (2019). Development of a Universal Influenza Vaccine. J. Immunol..

[146] Sandbrink J.B., Shattock R.J. (2020). RNA Vaccines: A Suitable Platform for Tackling Emerging Pandemics?. Front. Immunol..

[147] Liu C., Ginn H.M., Dejnirattisai W., Supasa P., Wang B., Tuekprakhon A., Nutalai R., Zhou D., Mentzer A.J., Zhao Y. (2021). Reduced neutralization of SARS-CoV-2 B.1.617 by vaccine and convalescent serum. Cell.

[148] Planas D., Veyer D., Baidaliuk A., Staropoli I., Guivel-Benhassine F., Rajah M.M., Planchais C., Porrot F., Robillard N., Puech J. (2021). Reduced sensitivity of SARS-CoV-2 variant Delta to antibody neutralization. Nature.

[149] Wu Y., Goplen N.P., Sun J. (2021). Aging and respiratory viral infection: From acute morbidity to chronic sequelae. Cell Biosci..

[150] Goplen N.P., Wu Y., Son Y.M., Li C., Wang Z., Cheon I.S., Jiang L., Zhu B., Ayasoufi K., Chini E.N. (2020). Tissue-resident CD8(+) T cells drive age-associated chronic lung sequelae after viral pneumonia. Sci. Immunol..

